# Validation of CORE-MD PMS Support Tool: A Novel Strategy for Aggregating Information from Notices of Failures to Support Medical Devices’ Post-Market Surveillance

**DOI:** 10.1007/s43441-022-00493-y

**Published:** 2023-01-18

**Authors:** Yijun Ren, Michele Bertoldi, Alan G. Fraser, Enrico Gianluca Caiani

**Affiliations:** 1 Department of Electronics, Information and Biomedical Engineering, Politecnico di Milano, Milan, Italy; 2 Department of Cardiology, University Hospital of Wales, Wales, CF14 4XW UK; 3 Istituto Auxologico Italiano IRCCS, Milan, Italy

**Keywords:** Medical device regulation, Natural language processing, Medical devices, Web scraping, Safety signal detection, Post-market surveillance

## Abstract

**Introduction:**

The EU Medical Device Regulation 2017/745 defines new rules for the certification and post-market surveillance of medical devices (MD), including an additional review by Expert Panels of clinical evaluation data for high-risk MD if reports and alerts suggest possibly associated increased risks. Within the EU-funded CORE-MD project, our aim was to develop a tool to support such process in which web-accessible safety notices (SN) are automatically retrieved and aggregated based on their specific MD categories and the European Medical Device Nomenclature (EMDN) classification by applying an Entity Resolution (ER) approach to enrich data integrating different sources. The performance of such approach was tested through a pilot study on the Italian data.

**Methods:**

Information relevant to 7622 SN from 2009 to 2021 was retrieved from the Italian Ministry of Health website by Web scraping. For incomplete EMDN data (68%), the MD best match was searched within a list of about 1.5 M MD on the Italian market, using Natural Language Processing techniques and pairwise ER. The performance of this approach was tested on the 2440 SN (32%) already provided with the EMDN code as reference standard.

**Results:**

The implemented ER method was able to correctly assign the correct manufacturer to the MD in each SN in 99% of the cases. Moreover, the correct EMDN code at level 1 was assigned in 2382 SN (97.62%), at level 2 in 2366 SN (96.97%) and at level 3 in 2329 SN (95.45%).

**Conclusion:**

The proposed approach was able to cope with the incompleteness of the publicly available data in the SN. In this way, grouping of SN relevant to a specific MD category/group/type could be used as possible sentinel for increased rates in reported serious incidents in high-risk MD.

## Introduction

The European Union (EU) Medical Device Regulation (MDR) 2017/745 that was published in the Official Journal of the EU on May 5th, 2017, and which has been effective since May 26th, 2021, is intended to ensure the safety and performance of medical devices (MDs) [1]. An important consequence of the new MDR 2017/745 is to resolve differences in the application of the previous Medical Device Directive 93/42/EEC between national systems, as a regulation is immediately applicable in all EU Member States. The main goals are to increase clinical evidence needed for MDs certification, as well as to improve their traceability and to strengthen post-market surveillance (PMS) [2].

According to Art. 2.1, a MD is defined as “any instrument, apparatus, appliance, software, implant, reagent, material or other article intended by the manufacturer to be used, alone or in combination, for human beings for one or more of the following specific medical purposes: diagnosis, prevention, monitoring”. MDs are classified into four classes based on the intended purpose of the devices and their inherent risk (Art. 51, MDR): class I (low risk), Class IIa (medium risk), Class IIb and Class III (high risk). A clinical investigation is mandatory for new devices in this last class (Art. 61.4, MDR). Additionally, as part of the conformity assessment procedure, for class III devices, the responsible notified body will be obliged to request a scrutiny (i.e. Clinical Evaluation Consultation Procedure, or CECP) of its Clinical Evaluation Assessment Report (CEAR) by an Expert Panel (EP), consisting of “advisors appointed by the Commission on the basis of their up-to-date clinical, scientific or technical expertise in the field” (Art. 106.3, MDR). The decision by the EP whether or not to proceed with the CECP needs to be taken within 21 days from the date of submission of the CEAR and is based on three decision criteria: the novelty of the device, any significantly adverse change in the benefit-risk profile, and a significant increase of serious incidents of a specific group/category [3]. The last two criteria seem strictly connected with the availability of a historical database of data, in which notices of failures during PMS are registered using harmonized nomenclature and standards among at least the EU member states. Safety signal detection is defined by the International Medical Device Regulators Forum (IMDRF) as “the process of determining patterns of association or unexpected occurrences that have the potential to impact decisions about patient management and/or to alter the known benefit-risk profile of a device” [4]. The spontaneous reporting systems, such as the FDA MAUDE, TGA DAEN, and the future EU EUDAMED (European database on medical devices), constitute a standard and required source for safety signal detection. While the main quantitative method for MD safety signal detection is disproportionality analysis [5], also others have been proposed, such as multivariate methods (change point analysis [6] and entity matching algorithm [7]), or methods based on the Data Extraction and Longitudinal Trend Analysis (DELTA) network. As a result, no agreement on the preferred methods for signal detection exists, and no gold standard for signal detection has been established so far [8].

As established by the MDR (Art. 33), the EUDAMED is being created. All the information relevant to actor registration, unique device identification (UDI) and device registration, notified bodies and certificates, clinical investigations and performance studies, vigilance and market surveillance will be conveyed in it. In principle, therefore, it will be possible to use EUDAMED to analyse safety signals, but there are delays in implementing its full operationality and it will take years to populate it with sufficient and properly collected data. Thus, a need has arisen to develop methods of aggregating data that can be applied to information that is already available, but incomplete and fragmented, not harmonized, from national sources [8].

Accordingly, in the context of the EU project CORE-MD (Coordinating Research and Evidence for Medical Devices [9]), we aimed at developing the CORE-MD PMS tool to automatically collect and display in an aggregated way accessible and official web-based historical content regarding to MD alerts and recalls, as a first step to apply methods for detecting safety signals for scientific analysis that may assist the EPs during their assessment of high-risk medical devices.

To do so, a first pilot feasibility study was conducted on the data available from the Italian Ministry of Health, to develop the methodology and minimum requirements needed to tackle the aforementioned problems and to validate the proposed strategy.

## Materials and Methods

**Figure 1:**
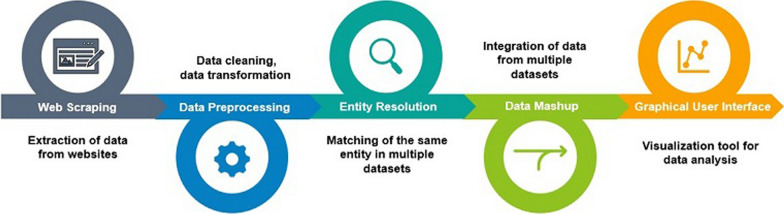
Methodological steps of our approach.

In Fig. 1, the main methodological steps involved in this work have been schematized, and in the following sections, a more detailed description for each of these steps will be given.

Briefly, data were first retrieved from the government websites of the Italian Ministry of Health by downloading the available Dataset of Devices (DoD), and by using Web scraping to get information about all safety notices (SN), thus, constituting the Dataset of Notices (DoN). After applying data cleaning and transformation to both DoD and DoN, the entity resolution technique was performed to match items in these two datasets, when a direct link was not already available. A mashup was then performed to integrate data and present it to the final user through a graphical interface.

## Data Sources

The Italian Ministry of Health supervises the Italian MD market and evaluates actions following incidents concerning MD. As part of its PMS, the manufacturer must report incidents regarding to any MD directly to the Ministry of Health, which generates the official SN from reports and makes all these SN publicly available through its official website, https://www.salute.gov.it/. We used the official website to generate the local DoN.

Another data source required by the proposed approach is constituted by the register of all MD (Dataset of devices, DoD) on the Italian market, which is also provided by the Italian Ministry of Health and publicly available at https://www.dati.salute.gov.it/dati/. It has been updated weekly since December 2011, and listed 1,474,975 devices on May 1, 2021. The DoD contains various items of information with respect to each MD, but for the purposes of this work, only the fields listed in Table 1 (with their description) were utilized.

**Table 1:** Useful information in dataset of devices

Variable	Description
Type	Type of MD: MD of class, IVD or assembled
Progressive DM/ASS	Progressive registration number attributed to MD
Manufacturer/assembler	Name of the manufacturer/assembler
Catalogue code	Identification of the MD according to the catalogue
Commercial name	Name of MD assigned by the manufacturer/assembler
Classification CND	Code of the “Classificazione Nazionale dei Dispositivi medici” corresponding to the European Medical Device Nomenclature

### The European Medical Device Nomenclature

In 2005, the Italian “Classificazione Nazionale dei Dispositivi medici” (CND) became the official Italian medical device classification and nomenclature. In March 2019, the CND was selected as the basis for the European Medical Device Nomenclature (EMDN) according to standards set out by the Medical Device Coordination Group [10]. The EMDN aims at supporting the functioning of EUDAMED, in accordance with MDR Art. 26 and Art. 23 of Regulation (EU) 2017/746 [11].

The CND code corresponding to the EMDN allows a clear knowledge of a sector composed by numerous and heterogenous products and to group them homogeneously due to its refined and hierarchical structure [12]. The code has an alphanumeric structure established in a multi-level hierarchical tree that helps cluster the MD in three main levels:

- Category: it represents the first hierarchical level with 22 anatomical or functional categories, each identified by a letter of the alphabet.
- Group: the second hierarchical level is identified by two-digit numbers from 01 to 99 for each category with a total of 146 anatomical or functional MD Groups.
- Type: the third hierarchical level expands into other levels, each identified by a couple of numbers where each type represents MD with a high affinity of use.

**Figure 2:**
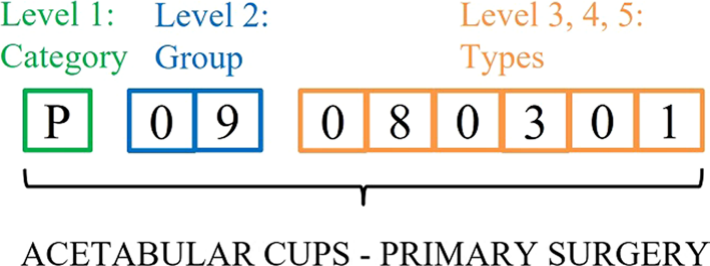
An example of EMDN code.

As a result, each MD is denoted by an alphanumeric code composed by a letter for the Category, two-digit numbers for the Group and other couples of numbers for the Type, up to a maximum of seven levels. Figure 2 shows an example of the EMDN code for acetabular cups.

## Web Scraping

Web scraping, known also as screen scraping or web harvesting, is the process of collecting a large amount of data from websites and transforming unstructured web data into metadata that can be stored and analysed [13]. Nowadays, Python represents the most popular programming language for Web scraping, as it can easily handle most of the processes and has various specific libraries to perform this task.

**Figure 3:**
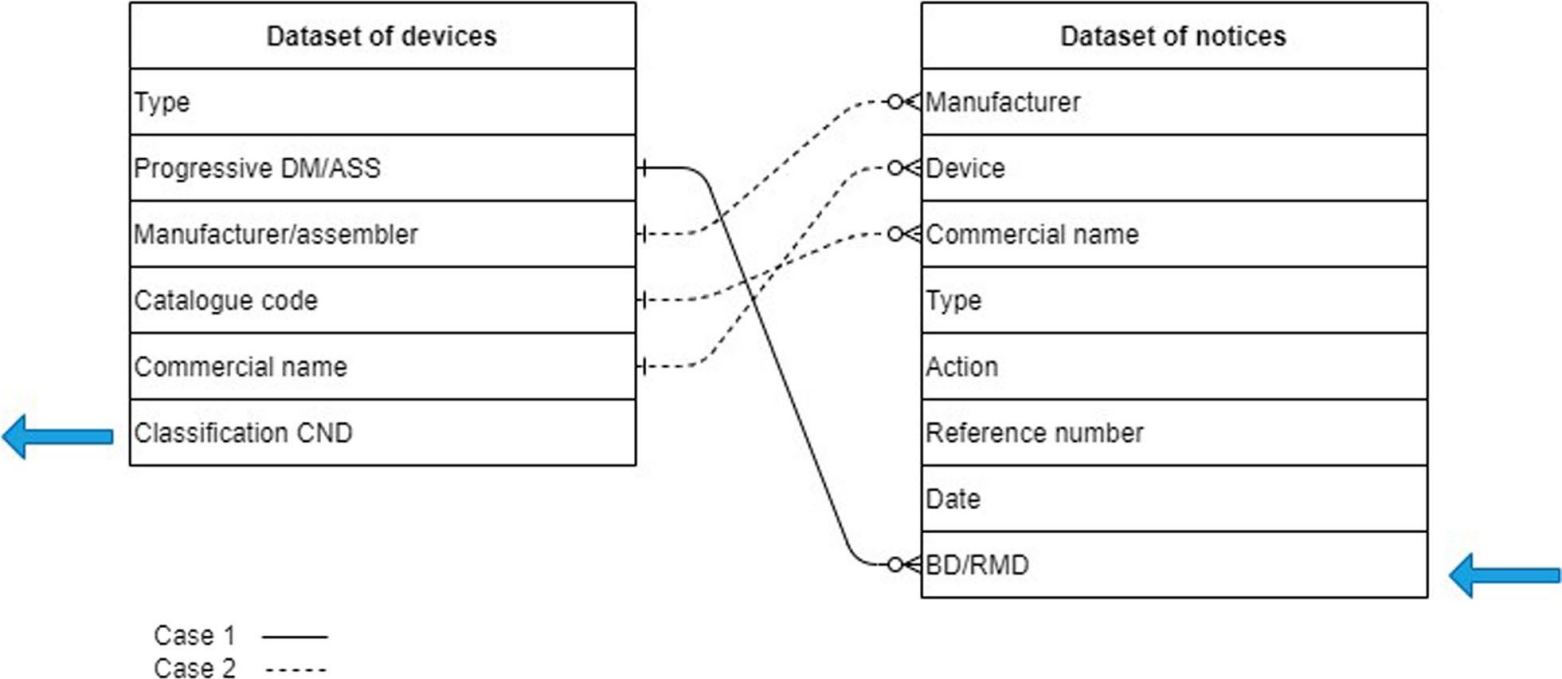
Correlated information presented in the two datasets and used for entity resolution.

In our application, Web scraping was needed as the information about PMS notices was only viewable as text on the website [14]. A bot was implemented to scrape the websites using the Python library Selenium, a suite of tools for automating web browsers [15]. ChromeDriver was used together with Selenium to automate the scraping on the Chrome browser [16]. The Selenium WebDriver drives the browser natively, as a user would, and can run either locally, as in our scenario, or on a remote machine using the Selenium server. In this way, the content of 763 webpages dating from 2009 to 2021, each containing 10 different notices (except for the last page), was retrieved by extracting the following fields from the html text (see Fig. 3, right):

- Manufacturer: name of the manufacturer which has reported the incident.
- Device: name of MD.
- Commercial name: name of MD, possibly with catalogue code or model specification.
- Type: type of MD, which can be active implantable medical device, *in vitro* diagnostics or MD.
- Action: the action of notice describes concisely the motivation of the safety notice by using a specific term such as recall, update, suspension of use, etc. Alternatively, it can consist of a short description of the report.
- Reference number: a unique number used to track down each safety notice reported by the website.
- Date: date of receipt.
- BD/RMD: directory number of the MD, which can be considered as a unique identification number of the MD on the Italian market.

## Data Preprocessing

Real-word data are usually susceptible to inconsistent data or missing data. Data preprocessing techniques, aiming to prepare more accurate data from raw data, have an important role in data analysis [17]. Also in our scenario, we used data cleaning techniques to address poor quality issues.

Since all the variables in DoD and in DoN are string variables, except for the variable *Date* in the DoN, a string-processing Python library, Natural Language Toolkit (NLTK), was used. It provides easy-to-use interfaces and a suite of text processing libraries for different text-mining tasks [18]. Another useful module for our analysis was the “re” module, providing a set of powerful regular expression-matching operations that allow checking whether a given string matches or contains a given pattern [19]. Using these modules, the following data transformation steps were performed: removal of punctuations, removal of extra spaces and special characters, and lowercasing (see Table 2). For the content of the *Manufacturer* field in the DoN and *Manufacturer/assembler* in the DoD, the word tokenization and Part-of-speech (POS) tag were also applied (see Table 2).

**Table 2:** The Explanation for procedures for the data preprocessing applied to the example “My pen is red!”

Procedure	Definition	Example^a^
Removal of punctuations	A process to remove punctuations within the text	“my pen is red”
Removal of extra spaces and special characters	A process to remove extra spaces and special characters such as “*” within the text	“My pen is red!”
Lowercasing	A process to convert all characters into lowercase	“my pen is red!”
Word tokenization	A process to split each sentence into words	[“My”, “pen”, “is”, “red”, “!”]
POS tag	A process to specify the words in the text for a particular part of a speech based on its definition and context	[(“My”, “PRP$”), (“pen”, “NN”), (“is”, “VBZ”), (“red”, “JJ”)]

^a^“PRP$” stands for possessive pronoun, NN” singular noun, “VBZ” verb in the present tense with 3rd person singular, and “JJ” adjective

## Entity Resolution

Entity resolution (ER), known also as record linkage or co-reference resolution, describes the problem of extracting, matching and resolving entity mentions in structured and unstructured data [20]. In other words, it is to determine whether items in different data sources refer to the same entity in the real world. The need for accurate and fast ER is growing, especially in this age of big data, where we are inundated with more and more data.

In this application scenario, the identification of the same MD in both datasets, DoN and DoD, is needed to associate the MD to the corresponding EMDN. The MD is uniquely identified by the variable *BD/RMD* in the DoN and by the *Progressive DM/ASS* in DoD. Two possible situations could be present:

- Case 1: the BD/RMD value was available for that record.
- Case 2: BD/RMD value was not present for that record.

In the first case, it was easy to connect the information in the two databases, so that the EMDN was automatically retrieved. In the second case, an ER approach was necessary to match the information of *Manufacturer* and *Device* fields in the DoN with *Manufacturer/assembler* and *Commercial name* in the DoD, as schematized in Fig. 3, in order to retrieve the corresponding EMDN Classification.

Several ER approaches are available: rule-based methods, pairwise classification, clustering approaches and probabilistic inference [20]. Since our problem was matching two entities independently from other mentions, pairwise ER was chosen. The aim was to compare two entity profiles at a time, and then decide whether these two records are matched or not, based on some similarity criteria. Due to computational complexity associated with the high number of devices in the DoD to be compared with every single notice in the DoN, the problem was tackled in two phases:

1. Identify similar manufacturers in the two datasets.
2. Identify similar devices among those records with similar manufacturers.

Note that the result of the first phase is crucial for the analysis and has a huge impact on the second one, as devices with different manufacturers will not be compared.

### Identification of the Similar Manufacturers

Two different methods were applied in the first phase: Named Entity Recognition (NER) and Cosine Similarity (COS).

Named Entity (NE) is defined as any named object, like a person or location. The NER represents a subproblem of information extraction with the aim of extracting NE in natural language text that refers to people, location, organization, companies etc. [21]. To find NE regarding to manufacturers in the two datasets, a pre-trained classifier provided by NLTK under the function *nltk.ne_chunk()* was applied. Once the NE relevant to *Manufacturer/assembler* in DoD and to *Manufacturer* in DoN were extracted, pairwise comparisons were performed considering two manufacturers as similar if and only if their corresponding extracted NE were the same.

The second applied approach was based on COS, which is easy to compute and results in values in a convenient range between 0 and 1 [22]. It uses the cosine between two vectors as a similarity measurement, regardless of their dimensions. First, the company’s suffix was removed (this step was absent in NER as the suffix was kept as informative to determine NE). Then, instead of using a simple Term Frequency (TF) vector that counts the frequency of appearance of each word in the text with dimension equal to the number of words in the entire document (union of all texts), the TF – Inverse Document Frequency (TFIDF) was applied, which evaluates also how relevant a word is in respect to the entire document [22]. The cosine similarity was computed to measure the similarity between two TFIDF vectors: they were considered as similar if the similarity score was equal or higher than 0.90 [23].

### Identify Similar Devices Among Those Records with Similar Manufacturers

After the identification of similar manufacturers by the step described above, in this second phase, the comparisons of the *Catalogue code* field in DoD with *Commercial name* in DoN, and of *Commercial name* in DoD with *Device* in DoN, need to be performed.

For this typical task of string-matching problem, which is a branch of Natural Language Processing (NLP), different approaches exist such as exact string-matching and approximate string-matching algorithms. In exact matching, all occurrences of the pattern P in text T are found [24]. However, if there is a difference due to spelling variation or misspelling, the exact matching might fail. Conversely, with the approximate string matching, all patterns that are “sufficiently like” P, or the N strings that are “most like” P are identified [25]. In this work, the approximate string matching was adopted, due to the inconsistencies in formatting and nomenclature in the two datasets [26]. In particular, the fuzzy string matching was implemented using the Python library Fuzzy-wuzzy [27]. In this way, rather than indicating only true or false, a similarity score from 0 to 100 was provided, by computing the Levenshtein distance to calculate the differences between samples. The Levenshtein distance computes the edit operations required to transform a string into another one (i.e. insertion, deletion or substitution) [28].

**Figure 4:**
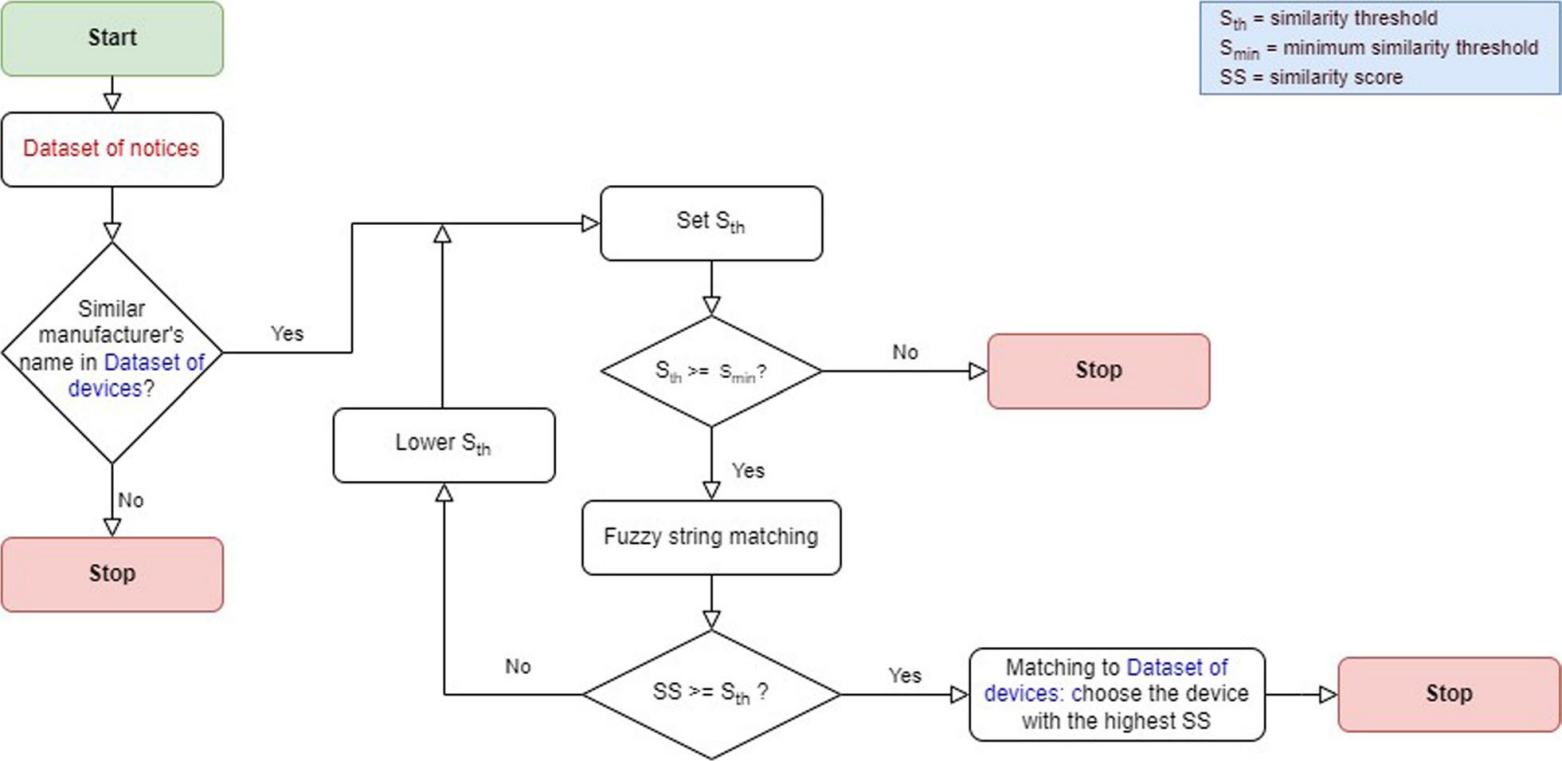
Workflow of the identification of the same MD in the two datasets.

The similarity threshold Sth was initially set equal to 95 to compare the information regarding to the devices among those with the previously matched manufacturers. If a matching with scores equal or higher than Sth exists, the device with the highest similarity score was selected; otherwise, the value of Sth is iteratively reduced with steps of 5 and the process is repeated until matching was found or Sth reached values lower than a minimum similarity threshold set equal to 60. In Fig. 4, the workflow for this process is represented.

As a result, if a matching was found between the devices in the DoN with those in the DoD, the corresponding *Classification CND* corresponding to the EMDN was retrieved and saved.

## Data Mashup

A mashup can be described as the integration of two or more homogeneous or heterogenous datasets into a unique Graphical User Interface (GUI) [29]. A mashup can also be made even from a single dataset, by combining its information in a way that has not been shown before, to extract deeper insights. To this aim, a GUI for the CORE-MD PMS tool was created displaying the SN in the DoN that were matched to some devices in the DoD, using the interactive data visualization, management, and analysis software Power BI tool (Microsoft) [30]. It offers data warehouse capabilities with several advantages: it connects different type of data sources, from Excel spreadsheets to databases; it offers a wide range of attractive visualizations and easy-to-process graphs; and it allows sharing insights using interactive dashboards [31].

**Figure 5:**
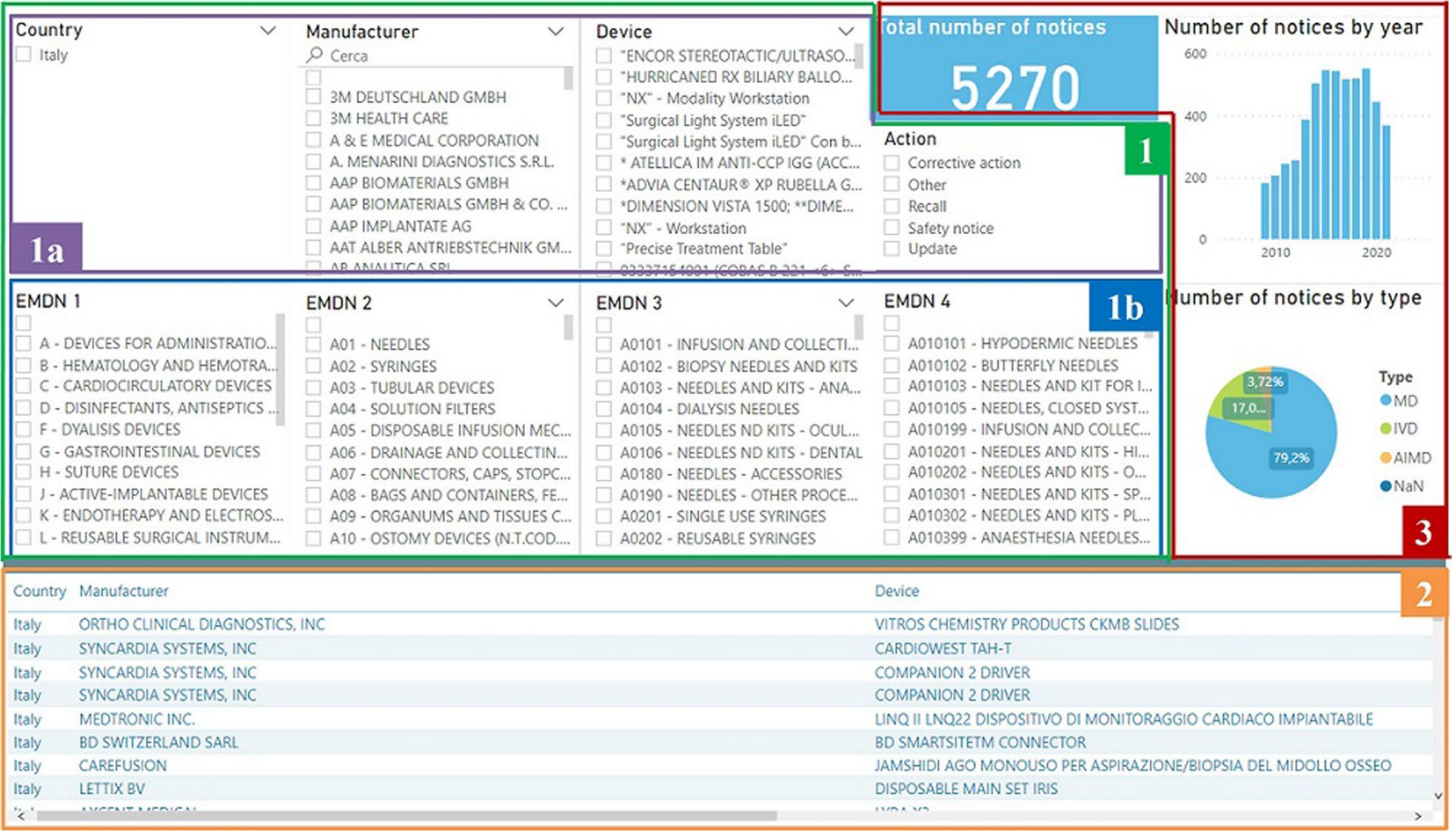
Graphical user interface developed by using Power BI.

Figure 5 shows the developed GUI, which can be divided into three major components. The first one, highlighted by the green box with number 1, is used to perform selections by clicking on one or more items of interest in visual objects. The second component, the orange box with number 2, shows all SN that satisfy requirements with the information about country, manufacturer, device, type of device, action, date and website that links to the original report. The third component, displayed as a red box with number 3 at the top right corner, provides a short summary of the filtered notices, including the total amount of notices that satisfy the conditions, the number of notices by year shown by the histogram and the number of notices by type of devices represented by the pie chart. By interacting with the histogram, SN can be limited to a particular time interval. The sub-component 1a is useful to filter information about SN, such as country, name of the manufacturer, name of the device and action, while the sub-component 1b helps users to select devices by using the EMDN classification from level 1 up to level 4.

## Model Validation

Several tests were performed to measure the performance of the proposed method in matching the MD in each SN of the DoN with the proper EMDN, using as reference criterion those records that presented a value in the *BD/RMD* field in the DoN, thus, allowing a direct retrieval of the corresponding EMDN from the DoD. In the following tests, the code identifier of the MD (defined here as *predicted BD/RMD*) represented by the field *Progressive DM/ASS* in the DoD chosen by the ER algorithm, as a result of the NER or the COS methods, was compared with the reference *BD/RMD* value to compute: (1) the number of SN in which the manufacturer was correctly matched to a device from the same manufacturer in the DoD; (2) the number of safety notices in which the *predicted BD/RMD* category of the devices was exactly matching the BD/RMD reference criterion, thus, resulting in the proper EMDN classification (up to EMDN level 4); (3) the performance of the ER algorithm was assessed, comparing the NER and COS methods, in assigning to the MD in each notice of the DoN the correct EMDN for each different level (up to EMDN level 4); (4) the ability in assigning to a MD the correct EMDN level despite the incorrect assignment of the BD/RMD code was evaluated, again considering the different levels of EMDN classification up to level 4.

The difference in performance between the NER and COS methods was evaluated by the mid-P version McNemar test, chosen as its power is very similar to the classical test version and it controls better the type I error rate [32].

## Results

If the *BD/RMD* field is available for a SN, then the device can be easily matched by searching for the same code represented by the *Progressive DM/ASS* in DoD. However, only about 32% of items (2440/7622) in DoN provided the corresponding *BD/RMD* code, so for the remaining devices the fuzzy string matching was needed. Applying the NER and COS methods to the DoN that included all the safety notices from 2009 to 06/08/2021, the number of devices that matched devices in DoD were

- Method NER: 6190 out of 7622 (81.2%, including those with *BD/RMD* code available).
- Method COS: 5270 out of 7622 (69.14%).

The time required for the fuzzy string-matching execution was about 5991 s (≈100 min) for NER and 1252 s (≈21 min) for COS.

To quantify the performance of these two methods, the tests described in the previous sections were applied by using as reference the subset of DoN constituted by 2440 items that already presented a value in the *BD/RMD* field.

**Figure 6:**
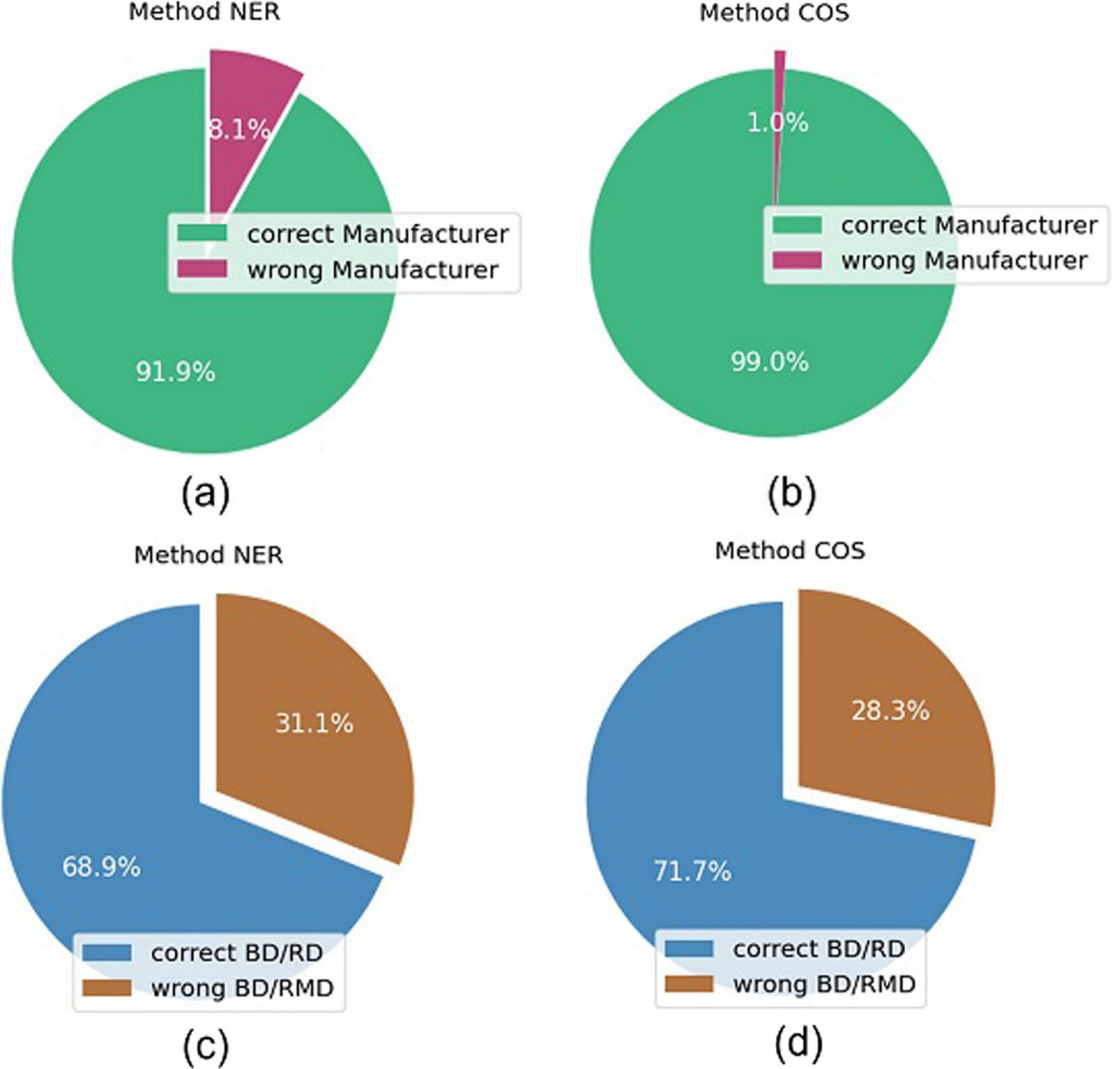
Percentage of correctly identified manufacturer or BD/RMD for the two tested approaches, NER and COS.

Figure 6 shows the results of the first and second tests. Figure 6a and b represents the percentage of correctly identified manufacturers using the NER and COS methods, respectively: with the latter, the correct manufacturer was identified in about 99% of the SN. Figure 6c and d shows the percentage of correctly identified BD/RMD for the two methods, resulting in 68.9% by NER and 71.7% by COS.

The result of the third test is shown in Table 3: the first EMDN level was correctly identified in 95.90% of the SN using the NER method and in 97.62% by the COS method, which provided better results for each considered level (up to level 4) of the EMDN with 94.67% of items correctly assigned to their EMDN code even at the fourth level.

**Table 3:** Percentage of correctly assigned EMDN levels for two approaches, NER and COS

EMDN level	Method NER (%)	Method COS (%)
1	95.90	97.62
2	94.75	96.97
3	92.95	95.45
4	91.97	94.67

To compute the fourth test, we focused on samples with incorrect assignment of BD/RMD code: there are 759 out of 2440 (31.1%) samples for the NER and 691 out of 2440 (28.3%) for the COS. The results of the fourth test are shown in Table 4: despite the wrong assignment of the BD/RMD code, the COS method was able to correctly assign the EMDN up to level 4 to 81.19% of samples with wrong BD/RMD, with higher performance than the NER (74.18%), and strong statistical significance (*p* = 8.79 × 10^–24^, McNemar test).

**Table 4:** Percentage of correctly assigned EMDN levels for two approaches, NER and COS, for samples that are assigned an incorrect RD/RMD

EMDN level	Method NER (%)	Method COS (%)
1	86.82	91.61
2	83.14	89.29
3	77.34	83.94
4	74.18	81.19

Since all information is retrievable from the government websites freely, and the main programming language used was Python, the work can be repeated and reproduced by following all steps described in Section “Materials and Methods” with the same input data (SNs published from 2009 to 06/08/2021). Otherwise, the results of different tests may vary slightly due to the inclusion of additional notices.

## Discussion

In this work, we developed a first prototype of the CORE-MD PMS tool to automatically collect and display in an aggregated way, accessible and official web-based historical content regarding to MD alerts and recalls. The CORE-MD PMS tool was designed as the first step to apply methods for detecting safety signals, and it was tested on the Italian data. The choice to refer to the Italian data was twofold: firstly, the SN and data regarding to MDs available on the Italian market were freely accessible from the government websites and they had been updated periodically since 2009; and secondly, the Italian nomenclature for MDs has now been adopted as EMDN for EUDAMED.

The fact that name of the manufacturer and of the device for each SN published on the website is clearly and separately provided, facilitated the entity resolution and guaranteed a high accuracy in the results. This characteristic is not granted in other government websites, such as, for example, France and Portugal, where the information regarding to manufacturers and devices is included in the title of the SN, so additional NLP techniques, or access to the associated.pdf document, would be required to disambiguate among them. Moreover, France has adopted a different nomenclature for MDs, the Global Medical Device Nomenclature, so the development of mapping between these nomenclatures would also be required.

To create a local database of the SN available on the website, web scraping was adopted; this approach is allowed, as data are publicly available and not copyrighted [33]. In fact, Europe recognizes the importance of text and data mining supported by web scraping, with Directive 2019/790 on Copyright and Related Rights in the Digital Single Market. In its Art. 4, it provides an exception from the rights of the database owner (copyright protection or sui generis protection) in case of “reproductions and extractions of lawfully accessible works and other subject matter for the purpose of the text and data mining” unless “the use of works (…) has not been expressly reserved by their rightholders in an appropriate manner” [34].

The adopted Selenium WebDriver scraping was able to examine 7622 html pages and extract the relevant information to generate the local DoN in about 3 h, which was utilized for the initial validation. However, the databases can be updated easily and automatically without additional computational effort, thanks to an automated check on the date of the last included information that allows updating it retrieving only the new data. As result, further monthly updates for SN using a separate routine will take less than 5 min.

To perform the dataset preprocessing, different techniques were applied, such as tokenization and POS tags. However, two common vocabulary normalization techniques such as stemming and lemmatization were not applied, as they were judged inappropriate for this application due to the fact that apart from the information about actions to take, the other text to be processed included only the names of the company and the MDs [22].

The library Fuzzy-wuzzy provides different functions to compare two strings; among them, the function *token_set_ration()* was used as it sorts the strings alphabetically and takes out the common tokens before calculating the distance, thus, overcoming possible problems relevant to the sequence of tokens and the repetition of words. Furthermore, it is the most helpful when the strings to be compared are of non-negligible difference in length, as was the case between *Catalogue code* in DoD and the longer *Commercial nam*e in DoN.

In this work, the minimum similarity threshold for the fuzzy string matching was set to 60. By varying this threshold within the range 60–100 with a step equal to 10, the relevant performance was assessed by comparing it with the percentage of correctly assigned EMDN levels from 1 to 4 (the third test presented in the paper). The best results were obtained with the minimum similarity threshold set to 60. The threshold for the cosine similarity was set to 0.9 as the relevant percentage of correctly identified manufacturers was the highest for the investigated range of values (0.9, 0.85, 0.8, 0.75).

From the different tests conducted, it is possible to state that the COS method was characterized by a higher performance than NER, and it required much less computational time. This could be explained by the fact that NER sometimes failed to extract information because of ambiguity and abbreviations [35]. In such cases, an empty string is returned, and the fuzzy string matching has to compute more than 1.4 M comparisons for the MD with this particular manufacturer, thus, slowing down the entire procedure.

The COS method is based on the cosine similarity between two strings, so either the strings are similar or dissimilar with respect to some threshold. As shown in Fig. 6, the COS method was able to correctly assign the BD/RMD code only for 71.7% of the samples in the validation; however, the method was more accurate than it could appear, since two identical MD could be registered with different Progressive registration numbers. In Table 5, an example of a SN with incorrect assignment of BD/RMD code is shown; despite this, the EMDN code was correctly assigned up to level 4 using COS. Further analysis highlighted that these two different BD/RMD codes referred to the same medical device in DoD, but it had been registered twice: the first time on March 10, 2018 with code 1,678,555, and then again on June 23, 2020 with code 1,967,116. This example shows that it is more important to have the EMDN levels correctly assigned rather than the BD/RMD code for the purpose of implementing safety signal detection on certain groups of devices. In this perspective, the potential of the COS method to assign the proper EMDN code up to the fourth level in 94.67% of the validating samples appears a very good performance.

**Table 5:** Example of a notice with incorrect assignment of BD/RMD, but with the EMDN code correctly assigned up to level 4

Variable	Real value	Predicted value by COS
BD/RMD	1,967,116	1,678,555
EMDN 1	Z—Medical equipment and related accessories and materials	Z—Medical equipment and related accessories and materials
EMDN 2	Z11—Bioimaging and radiotherapy instruments	Z11—Bioimaging and radiotherapy instruments
EMDN 3	Z1104 -Echographic instruments	Z1104 -Echographic instruments
EMDN 4	Z110401—Ultrasound scanners	Z110401—Ultrasound scanners

Cohen et al. [36] also proved that the COS method (also called TFIDF) showed to be the best among the token-based distance metrics considering strings as multisets of words, where SoftTFIDF, combination of TFIDF with the Jaro-Winkler string distance, resulted in the best overall distance measures but at expenses of larger matching computational time.

**Figure 7:**
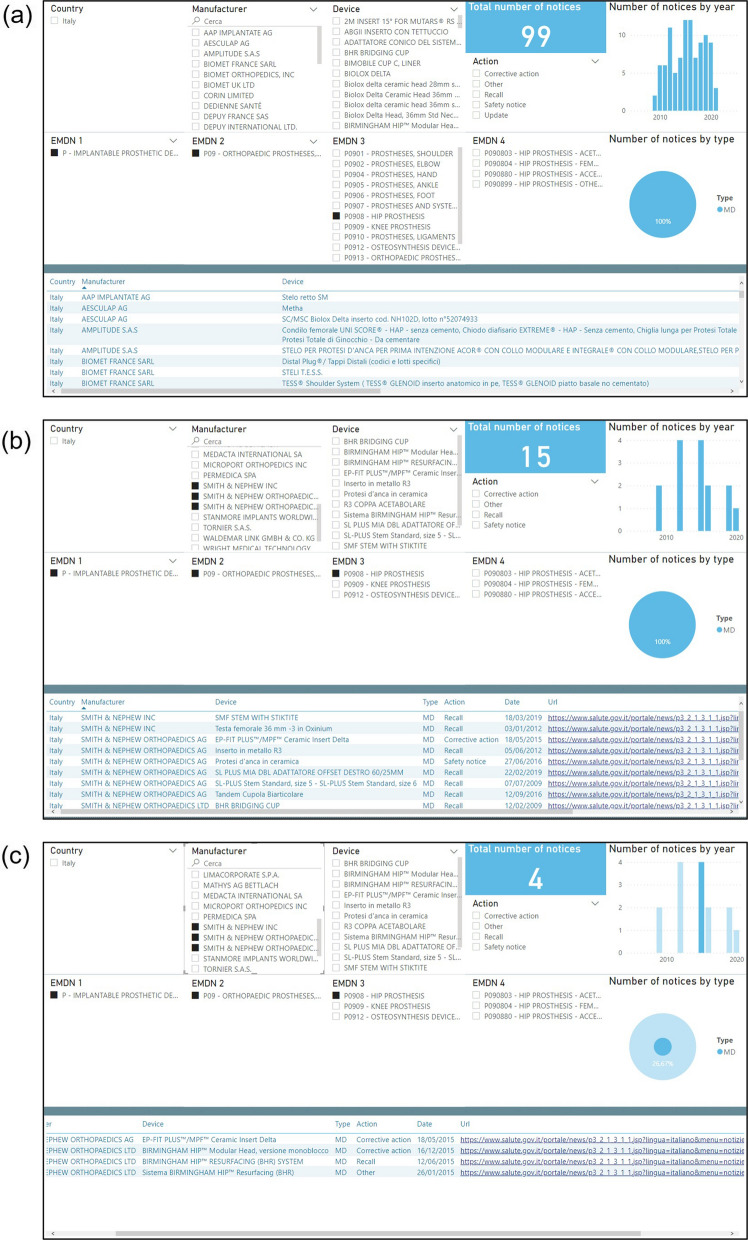
A case study of high-risk medical devices using the CORE-MD PMS tool. **a** querying for a specific category of MD, P0908, **b** for a specific company, and **c** for a specific year.

Figure 7 shows a case study of high-risk medical devices. Figure 7a displays the information extracted from the developed GUI for the category of P0908 (hip prosthesis). There are in total 99 SN related to this category from 2009 to 06/08/2021 in the Italian market, with the maximum value in 2015 and 2016 (12 SN). One of the largest orthopedic device companies in the world is Smith and Nephew. By selecting this company as shown in Fig. 7b, the GUI shows that there are about 15 out of 99 SN (15%) coming from this company. In addition, by interacting with the histogram, results can be limited to a smaller time interval as shown in Fig. 7c, which displays only those SN recorded in 2015 among the previously selected 15 notices. Note that 3 out of 4 SN are regarding the Birmingham Hip Resurfacing System. The query of this device in the search engine PubMed returned 35 related articles from 2004 to 2022 with the maximum number of published articles in 2016 (6 articles), which is doubled with respect to the previous year. So, an unusual increase in terms of the number of SN for a specific category of MD may be associated with an increase in related articles.

Like disproportionality analysis which requires only the number of events reported, the CORE-MD PMS tool is quick and inexpensive. While the former aims to identify the potential association between a specific device and a specific adverse event but would not help fix any data-related concerns, such as incomplete or non-validated data, our tool is able to transform unstructured information into structured data with the corresponding EMDN code. Moreover, as disproportionality analysis ignores useful information such as trends, change point analysis was proposed to determine changes within a product over time and can be applied to detect changes in the association between products and adverse events over time [6]. However, the latter suffers from underreporting or overreporting of events. Different from disproportionality analysis and change point analysis that should be performed separately for each device, the CORE-MD PMS tool is able to provide an overview of reported adverse events for each device, or even for each category of EMDN, in an automated way. Our tool shares some similarities with the entity matching algorithm [7], since both help determine which medical devices may be influenced based on new adverse events. In addition, our tool provides information on the category of devices. On other hand, methods based on the DELTA network, a multicenter prospective observational study designed to evaluate the safety of new cardiovascular devices, are considered automated safety surveillance tools. However, the DELTA network system is limited by the scope of the available clinical data and is subject to biases inherent to observational studies [37]. In addition, as the EUDAMED is still in development, once it is completed, it will not contain historical data. Our work is able to provide such historical information in a structured format.

Our study has several strengths. The databases can be updated easily and automatically without additional computational effort. The general framework of operations can be adopted and modified to aggregate data from other countries other than the Italian one; however, this possibility is dependent on the level of completeness of the available information from the official website, language, adopted MD nomenclature, thus, involving the specification and additional implementation of specific country-based solutions. The developed GUI allows for querying the aggregated data based on the user needs, retrieving both cumulative results (i.e. the number of SN over the year) and the list of the SNs, with the corresponding link to the original document. Our study also has some limitations. First, the information about the relationship between companies, such as subsidiaries or parent companies, is absent. Therefore, querying for a specific company’s name will return only the notices for that company and not those of possible company’ subsidiaries. Second, even if the IMDRF has proposed Adverse Event Terminology [38] to classify the root causes of the devices that appeared in the SNs, the classification of SNs based on this terminology was not performed in this work due to the absence of the necessary information, such as the summary of the reason of recall. To retrieve additional information, the related.pdf document, written in Italian and in different styles, with no standard format for reporting defined, should be opened and analysed.

## Conclusions

In this pilot study, we tackled the specific problem of aggregating publicly available data on safety notices which were characterized by incompleteness in the nomenclature reporting. The proposed solution resulting in the first version of the CORE-MD PMS tool was effective thanks to the availability of the list of medical devices on the Italian market, and to the proposed COS approach for entity resolution. Accordingly, data relevant to a specific MD category (up to the fourth level of the EMDN) can be selected and visualized throughout the developed graphical interface, as a first attempt to produce data usable to detect safety signals by published reports. As well, the provided information could be used day by day throughout the developed interface to check a specific MD already on the market, to evaluate whether there is any significant adverse change in the benefit-risk profile for that category in the PMS process, or to explore a certain MD category to evaluate the nature of failures in the attempt to design new solutions which minimize such problems.

This approach could constitute a valid solution to access historical data while EUDAMED would start to be populated with more standardized and complete information. While its feasibility was tested in only the Italian scenario, further research is currently ongoing in order to extend it to other national member states’ official websites, provided that data of safety notices and medical devices linked to the EMDN nomenclature are accessible.

## Data Availability

All data used is freely available and retrievable from the official government websites by using web-scraping techniques as described.

## Appendix

All acronyms used in this work are described in Table 6.

**Table 6:** List of acronyms in alphabetical order

Abbreviation	Definition
CECP	Clinical Evaluation Consultation Procedure
CECR	Clinical Evaluation Assessment Report
COS	Cosine similarity
CND	Classificazione Nazionale dei Dispositivi medici
CORE-MD	Coordinating Research and Evidence for Medical Devices
DAEN	Database of Adverse Event Notifications
DELTA	Extraction and Longitudinal Trend Analysis
DoD	Dataset of devices
DoN	Dataset of notices
EMDN	European Medical Device Nomenclature
ER	Entity resolution
EU	European Union
EUDAMED	European database on medical devices
EP	Expert Panel
FDA	Food and Drug Administration
MAUDE	Manufacturer and User Facility Device Experience
GUI	Graphical user interface
IMDRF	International Medical Device Regulators Forum
MD	Medical device
MDR	Medical Device Regulation
NE	Named entity
NER	Named entity recognition
NLP	Natural Language Processing
PMS	Post-market surveillance
SN	Safety notice
TF	Term Frequency
TFIDF	Term Frequency – Inverse Document Frequency
TGA	Therapeutic Goods Administration
UDI	Unique device identification
